# The modality of dialysis does not influence atheromatous vascular disease progression or cardiovascular outcomes in dialysis patients without previous cardiovascular disease

**DOI:** 10.1371/journal.pone.0186921

**Published:** 2017-11-02

**Authors:** Mercè Borràs Sans, Miguel Pérez-Fontán, Montserrat Martinez-Alonso, Auxiliadora Bajo, Àngels Betriu, José M. Valdivielso, Elvira Fernández, on behalf of NEFRONA INVESTIGATORS

**Affiliations:** 1 Department of Nephrology, University Hospital Arnau de Vilanova de Lleida, Lleida,Spain; 2 UDETMA Unitat de Detecció de Malalties Aterotrombòtiques, Lleida, Spain; 3 Vascular and Renal Translational Research Group, UDETMA, REDinREN del ISCIII, IRBLleida, Lleida, Spain; 4 Department of Nephrology, University Hospital A Coruña, A Coruña, Spain; 5 Department of Nephrology, University Hospital La Paz, REDinREN del ISCIII, Madrid, Spain; Nagoya University, JAPAN

## Abstract

**Background:**

There is limited and inconclusive information regarding the influence of the modality of renal replacement therapy on the atherosclerotic burden of patients on dialysis. The aim of this study was to compare the prevalence of asymptomatic atheromatous carotid disease, as also its rate of progression and cardiovascular outcomes, in two matched populations of patients treated with hemodialysis (HD) and peritoneal dialysis (PD).

**Methods:**

Following a prospective, observational and multicenter design, we compared 237 PD and 237 HD patients without previous cardiovascular disease, included in the NEFRONA study, and matched for age, sex, diabetes and time on dialysis. Carotid ultrasound study was performed at baseline and after two years of follow-up in 6 carotid territories. Atheromatous vascular disease (AVD) progression was defined as any increase in the number of territories with plaques after 2 years. Fatal and non fatal cardiovascular events were also recorded during 36-month of follow-up.

**Main results:**

At baseline, PD patients presented a worse general cardiovascular risk profile than HD patients. On the contrary, some markers of prevalent atherosclerotic disease (common carotid intima-media thickness and ankle-brachial index) were more favorable in PD patients. During follow-up, we observed no differences either in the rate of progression of atheromatous vascular disease (OR 1.78, 95% CI 0.80–4.06, p = 0.161) or in the incidence of cardiovascular events (OR 1.51, 95% CI 0.85–2.66, p = 0.159), according to the modality of dialysis.

**Conclusion:**

Dialysis modality did not impact on atherosclerotic carotid disease progression or cardiovascular outcomes, in two groups of patients treated with PD or HD.

## Introduction

Cardiovascular disease (CVD) is the leading cause of mortality in patients with end stage renal disease (ESRD) [1,2]. Available evidence suggests that atherosclerosis is a primary contributor to this outcome, although non-atherosclerotic CVD, including volume overload and left ventricular hypertrophy, may also play a significant role in the increased CV mortality observed in these patients. Patients on dialysis present a high burden of traditional risks factors for CVD, including dyslipemia, diabetes mellitus and hypertension, usually present at the initiation of therapy. In addition, many nontraditional risk factors for this condition, including hyperparathyroidism, hyperfibrinogenemia, hyperhomocysteinemia, hypoalbuminemia and others [3,4] are also very prevalent. The modality of dialysis itself–hemodialysis (HD) or peritoneal dialysis (PD)–could also have a differentiated effect on general and specific CV risk factors, and also on the progression of atherosclerotic disease (AD). If this was the case, the effect should be more pronounced as time on dialysis increases.

High-resolution carotid ultrasonography (cUS) is a reliable and relatively simple instrument for the study of atherosclerotic vascular disease. Increased carotid intima media thickness (cIMT) and the presence of calcified and non calcified plaques are strong predictors of CV events in the general population [5–7], and have been claimed to portend similar outcomes in patients with ESRD treated with HD [8–10] or PD [10].

There is limited and inconclusive information regarding the compared atherosclerotic burden according to the modality of dialysis, as estimated by cUS [11–13]. Cross-sectional studies may provide information on this question, but the risk of selection biases hampers the interpretation of the results. A longitudinal design could provide more reliable information but, to our knowledge, this approach has not been undertaken, so far.

The aim of the present study is to compare the risks of progression of atheromatous arterial disease and to assess risk factors of incident cardiovascular outcomes, in two matched populations of patients treated with HD and PD, respectively.

## Material and methods

### Study design and participants

The NEFRONA project is a Spanish multicentric, observational, prospective study, designed to investigate the atherosclerotic burden of patients with chronic kidney disease (CKD), including relatively large samples of patients with ESRD treated with HD or PD. The general design and objectives of NEFRONA have been reported in detail [14,15]. In summary, 2445 CKD patients (of whom 688 were prevalent patients on dialysis), 18–75 years of age, were enrolled from 81 Spanish hospitals between October 2010 and June 2012, with a scheduled follow-up visit after 24 months. A main inclusion criterion was the absence of overt atherosclerotic disease at the start of follow-up. Consequently, patients who had stenotic carotid plaques or ankle-brachial index (ABI) <0.7 at baseline evaluation were excluded from the follow-up visit. Patients who suffered a CV event, received a renal allograft or died after the first ultrasound exploration, were also excluded from the second exploration.

The objective of the present study was to compare the progression of carotid artery atherosclerotic disease and assess risk factor of incident cardiovascular events in patients treated with PD and HD. Given the usual mismatches when these two types of patients are compared, we created two groups of patients. The NEFRONA study included a total of 237 PD patients (all of them included in this study) together with 451 HD patients. A selection of 237 HD patients matched by age, sex, diabetes and time on dialysis was performed from the group of HD patients in order to get comparable groups, with the ultimate aim of reducing the bias risk.

The study protocol was approved by the ethics committee of University Hospital Arnau de Vilanova, Lleida, Spain, and written informed consent was requested to all participants. The study complied with the principles of the Declaration of Helsinki.

### Clinical and biochemical data

At the time of recruitment, information about current health status, medical history, former cardiovascular risk factors and drug use was obtained. A physical examination was performed, including in anthropometric measures, standard vital tests and ABI measurement as previously described [16]. A pathological ABI was described as ≤0.9 or ≥1.4. Biochemical data were obtained from a routine blood test performed within three months of the vascular study. For HD patients, blood samples were retrieved at the start of the second session of the week. Parathyroid hormone (PTH) level was standardized using a recognized conversion method [17] to overcome inter-method variability between different centers. Determinations of high-sensitivity C reactive protein (hsCRP), 25hydroxy-vitamin D and 1.25hydroxy-vitamin D were performed in a centralized laboratory, to avoid variability among methods.

### Carotid ultrasound (cUS)

B-mode ultrasound of the carotid arteries was performed using the Vivid BT09 device (General Electric Instruments, Freiburg, Germany), with the help of 6–13 MHz broadband linear array probes. The measurement of cIMT and the analysis on presence of atheromatous plaques was performed by a single reader in a blinded fashion, using the semi-automatic software EchoPAC Dimension (General Electric Healthcare, Harten, Norway). We previously assessed the quality of the reading and the intraobserver variability, using a sample of 20 individuals in whom estimations were performed 3 to 5 times at different days. A kappa coefficient of 1 was obtained, indicating an optimal intraobserver reliability.

US imaging was performed for both carotid arteries with the subjects in a supine position and the head turned 45° contralateral to the side of the probe. cIMT was measured in the last centimeter of the far wall of the common carotid artery, the bulb section and, finally, the first centimeter of the internal carotid artery. Measurements were made in plaque-free arterial segments. The presence of atheromatous plaques in each of the mentioned points was defined by a cIMT ≥ 1.5 mm protruding to the lumen, following the recommendations of the ASE Consensus Statement [18] and the Mannheim cIMT Consensus report [19].

We created a carotid plaque score, which resulted from the addition of the number of points scrutinized (n = 6), including common, bulb and internal carotid arteries in each side in which at least one plaque was detected. Thus, the range of the score extended between 0 (no plaques) to 6 (all sites examined with plaque). We defined progression of AD over the two-year study span as any increase in the number of territories with plaque, when compared with to the baseline visit, as previously reported in the MESA study [20].

### Study variables and data analysis

The main study variable was dialysis modality classified as HD (including hospital, satellite, and home-based HD) or PD (including continuous ambulatory PD and automated PD) according to the dialysis at the study inclusion. First main outcome variable was progression of AVD defined according to cUS results by any increase in the number of territories with plaque at the c-US two-year study when compared with the baseline study, measured by the plaque score. The second main outcome variable was presentation of fatal and non-fatal CV events during a minimum of 36 month follow up. CV events were defined according to the International Classification of Diseases, Ninth Revision, Clinical Modification (ICD9-CM) which includes unstable angina, myocardial infarction, transient ischemic attack, cerebrovascular accident, congestive heart failure, arrhythmia, peripheral artery disease or amputation for vascular disease and aorta aneurisma [14]. Control variables included demographic, clinical, biochemical and prescription factors depicted in Table 1 (all included patients) and Table 2 (patients with 2-years follow-up).

**Table 1:** Main Baseline characteristics according to dialysis modality.

	PD^a^ patients	HD^b^ patients	p
	(n = 237)	(n = 237)	
Males (%)	137 (58)	146 (61.8)	0.45
Age (years)	52 [42;63]	55 [43;63]	0.11
Smoker (former/current) (%)	135 (57)	137 (58)	0.92
Diabetes (%)	43 (18.1)	43 (18.1)	1
Hypertension (%)	219 (92.4)	199 (84)	0.007
Dyslipidemia (%)	153 (64.6)	123 (47.7)	<0.001
Etiology of renal disease (%):			0.670
Diabetic nephropathy	25 (10.5)	27 (11.4)	
Vascular disease	23 (9.7)	29 (12.2)	
Others	189 (79.7)	181 (76.4)	
Dialysis time (months)	13.7 [6.2;28.9]	14.3 [6.41;28.6]	0.91
Body mass index (kg/m^2^)	26.4 [23.3;29.2]	25.3 [23;28.6]	0.23
Systolic Blood Pressure (mmHg)	140 [129;159]	135 [120;151]	0.001
Diastolic Blood Pressure (mm Hg)	83 [77;94]	80 [70;88]	<0.001
Pulse Pressure (mmHg)	56 [45;69]	55 [46;63]	0.53
Serum glucose (mmol/L)	5.05 [2.96;6.10]	5.05 [4.56;6.10]	0.838
Urea (mmol/L)	21.5 [17.6;25.8]	20.8 [15.8;25]	0.07
Creatinine (μmol/L)	682.5 [519.8;884]	727.5 [574.6;884]	0.1
Total colesterol (mmol/L)	4.58 [3.99;5.20]	3.91 [3.37;4.53]	<0.001
HDL-cholesterol (mmol/L)	1.22 [1.01;1.48]	1.09 [0.90;1.29]	<0.001
LDL-cholesterol (mmol/L)	2.66 [2.07;5.15]	2.07 [1.63;2.64]	<0.001
Triglycerides (mmol/L)	1.34 [1.07;1.87]	1.41 [1.03;1.92]	0.75
Serum uric acid (μmol/L)	345.1 [303.4;401.6]	362.9 [321.3;425.4]	0.006
hs C-Reactive Protein (nmol/L)	19 [8.95;49.9]	22.47 [10.09;58.66]	0.23
Albumin (mol/L)	0.59 [0.54;0.62]	0.59 [0.54;0.64]	0.025
Hemoglobin (mmol/L)	1.88 [1.75;2]	1.81 [1.67;1.94]	<0.001
Corrected calcium (mmol/L)	2.3 [2.20;2.42]	2.26 [2.16;2.36]	<0.001
Phosphate (mmol/L)	1.6 [1.35;1.84]	1.54 [1.25;1.79]	0.035
iPTH (pmolL)	22.7 [14.9;35.4]	25.7 [14.3;36.4]	0.23
25-hydroxy-vitamin D (nmol/L)	28.9 [20.9;41.2]	37.4 [26.4;48.7]	<0.001
1-25-hydroxy-vitamin D (pmol/L)	13.6 [9.72;19.9]	13.8 [9.31;22.3]	0.54
Treatments			
Antihypertensive (%):	214 (90.3)	159 (67.1)	<0.001
ACEI^c^ (%)	66 (27.8)	41 (17.3)	0.008
ARBs^d^ (%)	111 (46.8)	54 (22.8)	<0.001
Diuretics (%)	128 (54)	45 (19)	<0.001
Statins (%)	137 (57.8)	110 (46.4)	0.017
Phosphate binders (%):	198 (83.5)	189 (79.7)	0.343
Binders without Ca^e^ (%)	126 (53.2)	138(58.2)	0.309
Binders with Ca^e^ (%)	42 (47.2)	40 (47.1)	1
Ca^e^ intake (binders) (gr/day)	1.5 [1;2]	1.65 [1;3]	0.027
Calcitriol/Paricalcitol (%)	97 (40.9)	122 (51.5)	0.027
Calcifedol /%)			
Cholecalciferol (%)	27 (11.4)	8 (3.38)	0.002
Cinacalcet (%)	11(4.64)	8 (3.38)	0.640
Antiplatelet drugs (%):	27 (24.1)	68 (28.7)	0.297
ESA^f^(%)	181(76.4)	198 (83.5)	0.066
Renal transplantation centre (%)	88 (37.1)	62 (26.2)	0.014
Plaque presence (%)	123 (51.9)	143 (60.3)	0.08
Number of territories with plaque	2 [1.0;3.0]	2 [1.0;3.0]	0.285
cIMT^g^ (mm)	0.65 [0.56;0.79]	0.70 [0.6;0.84]	0.009
Ankle-Brachial index (%)			
ABI ≤ 0.9	37 (15.7)	23 (9.91)	0.08
ABI >0.9-<1.4	175 (74.5)	144 (62.1)	0.005
ABI ≥ 1.4	23 (9.79)	65 (28)	<0.001

Data are presented as median [interquartile range], mean (standard deviation) or n (%).

^a^ Peritoneal dialysis

^b^Hemodialysis

^c^ Angiotensin converting enzyme inhibitors

^d^ Angiotensin II receptor blockers

^e^ Calcium

^f^ Erythropoiesis stimulating agent1

^g^ Common carotid artery intima media thickness

**Table 2:** Main Baseline characteristics of patients with 2-years follow-up according to dialysis modality.

	PD^a^ patients	HD^b^ patients	p
	(n = 82)	(n = 85)	
Males (%)	47 (57.3)	48 (52.2)	0.6
Age (years)	53 [42.5;63]	55 [44.8;64]	0.39
Smoker (former/current) (%)	49 (59.8)	48 (52.2)	0.9
Diabetes (%)	14 (17.1)	14 (15.2)	0.9
**Hypertension (%)**	75 (91.5)	79 (86)	0.359
**Dyslipidemia (%)**	58 (70.7)	55 (59.8)	0.176
Etiology of renal disease (%):			0.670
Diabetic nephropathy	7 (8.54)	8 (8.7)	
Vascular disease	8 (9.76)	13 (14.1)	
Others	67 (81.7)	71 (77.2)	
Dialysis time (months)	10.5 [4.46;19.2]	11.5 [5.82;25.8]	0.131
Body mass index (kg/m^2^)	26.4 [23.3;28.4]	26.1 [23;4.32.2]	0.446
Systolic Blood Pressure (mmHg)	145 (24.4)	138 (21.8)	0.03
Diastolic Blood Pressure (mm Hg)	87.5 (12.2)	78.8 (13.3)	<0.001
Pulse Pressure (mmHg)	56 [44;69]	56.5 [47;70.2]	0.579
Serum glucose (mmol/L)	5.16 [4.72;5.61]	5.23 [4.55;6.05]	0.931
Urea (mmol/L)	22.11 (6.76)	20.31 (6.13)	0.053
Creatinine (μmol/L)	596.7 [468.5;792.1]	676.3 [583.4;837.2]	0.815
Total colesterol (mmol/L)	4.75 [4.2;5.43]	3.9 [3.2;4.45]	0.258
HDL-cholesterol (mmol/L)	1.21 [1;1.52]	1.1 [0.88;1.21]	0.005
LDL-cholesterol (mmol/L)	3.46 [2.28;3.05]	3.36 [2.56;4.4]	<0.001
Triglycerides (mmol/L)	1.53 [1.09;2.02]	1.48 [1.13;1.94]	0.940
Serum uric acid (μmol/L)	350.4 (72.6)	369.4 (70.78)	0.093
hs C-Reactive Protein (nmol/L)	26.28 [10;61.24]	24 [10.47;62.48]	0.815
Albumin (mol/L)	0.59 [0.53;0.62]	0.59 [0.54;0.64]	0.471
Hemoglobin (mmol/L)	1.89 (0.22)	1.78 (0.22)	0.002
Corrected calcium (mmol/L)	2.32 [2.22;2.4]	2.25 [2.16;2.32]	0.031
Phosphate (mmol/L)	1.52[1.32;1.81]	1.55 [1.29;1.81]	0.965
iPTH (pmolL)	21.1 [15.48;34.25]	26.51 [16.2;41]	0.182
25-hydroxy-vitamin D (nmol/L)	30.5 [21.75;43.75]	35.5 [27;48]	0.014
1-25-hydroxy-vitamin D (pmol/L)	15.45 [10.3;22.7]	14.4 [10.15;25.75]	0.862
Treatments			
Antihypertensive (%):	72 (87.8)	60 (65.2)	0.001
ACEI^c^ (%)	20 (24.4)	15 (16.3)	0.255
ARBs^d^ (%)	37 (45.1)	25 (27.2)	0.021
Diuretics (%)	27 (32.6)	28 (32.9)	0.837
Statins (%)	48 (58.3)	50 (54.3)	0.687
Phosphate binders (%):	66 (80.5)	68 (73.9)	0.396
Binders without Ca^e^ (%)	38 (46.3)	48(52.2)	0.538
Binders with Ca^e^ (%)	42 (47.2)	40 (47.1)	1
Ca^e^ intake (binders) (gr/day)	1 [1;1.5]	1.5 [1;2.5]	0.03
Calcitriol/Paricalcitol (%)	34 (41.5)	44 (47.8)	0.490
Calcifedol /%)	8 (9.76)	5 (5.43)	0.428
Cholecalciferol (%)	2 (2.44)	4 (4.35)	0.685
Cinacalcet (%)	18 (22)	24 (26.1)	0.646
Antiplatelet drugs (%):	22 (26.8)	27 (29.3)	0.842
ESA^f^ (%)	62 (75.6)	77 (83.7)	0.255
Renal transplantation centre (%)	27 (32.9)	20 (21.7)	0.137
Plaque presence (%)	44 (53.7)	53 (57.6)	0.711
Number of territories with plaque	2 [1.0;2.0]	2 [1.0;3.0]	0.149
cIMT^g^ (mm)	0.65 [0.57;0.75]	0.72 [0.62;0.86]	0.005
Ankle-Brachial index (%)			<0.001
ABI ≤0.9	10 (12.2)	7 (7.78)	
ABI >0.9-<1.4	66 (80.5)	55 (61.1)	
ABI ≥ 1.4	6 (7.32)	21 (31.1)	

Data are presented as median [interquartile range], mean (standard deviation) or n (%).

^a^ Peritoneal dialysis

^b^ Hemodialysis

^c^ Angiotensin converting enzyme inhibitors

^d^ Angiotensin II receptor blockers

^e^ Calcium

^f^ Erythropoiesis stimulating agents

^g^ Common carotid artery intima media thickness

### Statistical analysis

Summary measures included median and interquartile intervals for quantitative variables. Qualitative variables were summarized with absolute and relative frequencies and we used the chi-squared test (or exact Fisher test when the expected frequencies was less than 5 in some cell) for comparisons between groups. We used Mann-Whitney’s test to compare quantitative variables between two groups, and Kruskal-Wallis’ test to compare three or more groups. A multivariate logistic regression model for atherosclerotic disease progression was fitted, including all significant covariates (according to the likelihood ratio test) and recoding those quantitative variables according to a cutoff value improving discrimination. Interactions were assessed as well as model’s calibration and discrimination. The analysis of risk factors of incident cardiovascular events was based on hazard ratio estimations based on log-rank test for qualitative variables and Cox’s proportional hazards regression for quantitative variables. A final multivariate Cox’s proportional hazards regression model was fitted, including the assessment of interactions and the performance of a test of the proportional hazards assumption.

## Results

### Baseline characteristics

A total of 237 PD and 237 HD patients were included. Tables 1 and 2 show the main baseline patients' characteristics, according to dialysis modality. Table 1 with all included patients and Table 2 with the patients with 2-years follow-up. No differences were observed in plaque presence and number of carotid territories (plaque score) when baseline atherosclerotic burden was compared between PD and HD patients. However, HD patients had higher cIMT and more pathological ABI: fewer patients had a normal ABI and more individuals showed an increased ABI compared to PD patients.

### Outcomes

Of the 474 dialysis patients included in the baseline analysis, 214 (45.1%) received a renal allograft during the ensuing 36 months. PD patients were significantly more likely to be transplanted than HD patients (50.2% *vs* 40.1%; p = 0.03). Fourteen patients died from non CV diseases; this outcome was less likely in PD patients (1,9%) than in HD patients (8.4%)(p = 0.03). Fifty-one (10.7%) patients either presented a CV event or died from CV disease, without significant differences between PD (11,8%) and HD patients (9,7%)(p = 0.13). Thirty two (6.5%) patients were lost to follow-up (p = 0.28). Finally, 21 (4.4%) patients did not undergo a second cUS, because they had stenotic carotid plaque or ABI <0.7 at baseline (n = 20), or because they had a maximal plaque score at baseline (n = 1), which prevented any possibility of score progression.

### Analysis of atheromatous vascular disease progression after two years

Of the 174 patients with a full 24-month evaluation, 80 (34%) were treated with PD, and 94 (40%) with HD. The proportion of patients presenting at least one carotid plaque increased significantly after 24 months of follow-up, from 56.1% to 70.3% (p = 0.0001). Progression of the lesions occurred in 51.1% of the patients, and the mean number of territories affected by plaque increased from 1.30 ±1.50 to 1.98 ±1.78 (p<0.01).

Univariate analysis of baseline potential risk factors for AVD progression is presented in Table 3. Patients with AVD progression were older, with more prevalence of diabetes, higher levels of c-Reactive Protein and lower levels of 25-hydroxy-vitamin D.

**Table 3:** Baseline factors associated with progression of atheromatous vascular disease (AVD) in all patients.

	AVD^a^ progression	Non AVD progression	p
	(n = 89)	(n = 85)	
Males (%)	50 (56.2)	45 (52.9)	0.78
Age (years)	57 [48;65]	50 [39;62]	0.004
Smoker (former/current) (%)	53 (59.6)	44 (51.8)	0.38
Diabetes (%)	20 (22.5)	8 (9.41)	0.033
Hypertension (%)	79 (88.8)	75 (88.2)	1
Dyslipidemia (%)	59 (66.3)	54 (63.5)	0.824
Etiology of renal disease (%):			0.165
Diabetic nephropathy	25 (10.5)	27 (11.4)	
Vascular disease	23 (9.7)	29 (12.2)	
Others	69 (77.5)	69 (81.2)	
Dialysis time (months)	10.2 [4.4;23.2]	11.8 [6.47;22.5]	0.27
Body mass index (kg/m^2^)	27 [23.6;30.7]	26.1 [23.1;28.8]	0.156
Systolic Blood Pressure (mmHg)	142 (24.3)	141 (24.3)	0.735
Diastolic Blood Pressure (mm Hg)	82 (13.5)	83.9 (13.5)	0.343
Pulse Pressure (mmHg)	60 [44;74]	56 [45;66]	0.326
Serum glucose (mmol/L)	5.18 [4.54;6.10]	5.05 [4.49;5.66]	0.157
Urea (mmol/L)	18.3 (5.25)	18.3 (5.21)	0.984
Creatinine (μmol/L)	610 [486.2;797.4]	662.1 [557.8;839.8]	0.089
Total colesterol (mmol/L)	4.14 [3.63;4.89]	4.45 [3.57;5.33]	0.258
HDL-cholesterol (mmol/L)	1.10 [0.93;1.39]	1.16 [0.96;1.37]	0.561
LDL-cholesterol (mmol/L)	2.30 [1.85;2.82]	2.61 [1.81;2.98]	0.264
Triglycerides (mmol/L)	1.51 [1.11;2.10]	1.38 [1.07;1.92]	0.528
Serum uric acid (μmol/L)	370.5 (72.6)	350.4 (70.8)	0.084
hs C-Reactive Protein (nmol/L)	37.7 [13.8;65.9]	18.5 [8.4;54.2]	0.04
Albumin (mol/L)	0.59 [0.0.53;0.63]	0.59 [0.54;0.62]	0.613
Hemoglobin (mmol/L)	1.83 (0.25)	1.83 (0.21)	0.962
Corrected calcium (mmol/L)	2.31 [2.19;2.4]	2.26 [2.12;2.37]	0.419
Phosphate (mmol/L)	1.52 [1.26;1.81]	1.55 [1.36;1.84]	0.292
iPTH (pmolL)	24.7 [15.9;38.1]	23.3 [15.27;36.7]	0.586
25-hydroxy-vitamin D (nmol/L)	30.9 [22.8;42.9]	36.4 [26.7;48.7]	0.043
1-25-hydroxy-vitamin D (pmol/L)	14.9 [10.0;24.5]	13.6 [9.7;22.3]	0.602
Treatments			
Antihypertensive (%):	68 (76.4)	64 (75.3)	1
ACEI^b^ (%)	18 (20.2)	17 (20)	1
ARBs^c^ (%)	29 (32.6)	33 (38.8)	0.483
Diuretics (%)	27 (32.6)	28 (32.9)	0.837
Statins (%)	51 (57.3)	47 (55.3)	0.9
Phosphate binders (%):	68 (76.4)	66 (77.6)	0.98
Binders without Ca^d^ (%)	39 (43.8)	47 (55.3)	0.173
Binders with Ca^d^ (%)	42 (47.2)	40 (47.1)	1
Ca^d^ intake (binders) (gr/day)	1 [1;1.5]	1.5 [1;2.5]	
Calcitriol/Paricalcitol (%)	45 (50.6)	33 (38.8)	0.16
Calcifedol /%)	9 (10.1)	4 (4.71)	0.286
Cholecalciferol (%)	3 (3.37)	3 (3.53)	1
Cinacalcet (%)	19 (21.3)	23 (27.1)	0.482
Antiplatelet drugs (%):	27 (30.3)	22 (25.9)	0.628
ESA^e^ (%)	66 (74.2)	73 (85.9)	0.082
Renal transplantation centre (%)	22 (24.7)	25 (29.4)	0.599
Plaque at baseline (%)	64 (71.9)	33 (33.8)	<0.001
Number of territories with plaque	2 [1.0;3.0]	2 [1.0;3.0]	0.398
cIMT^f^ (mm)	0.73 [0.65;0.89]	0.64 [0.56;0.74]	<0.001
Ankle-Brachial index (%)			
ABI ≤0.9	11 (12.5)	6 (7.14)	0.357
ABI >0.9-<1.4	57 (64.8)	64 (76.2)	0.141
ABI ≥ 1.4	20 (22.7)	14 (16.7)	0.420
**Dialysis modality**:			**0.437**
**Hemodialysis**	**44 (49.4)**	**48 (56.5)**	
**Peritoneal dialysis**	**45 (50.6)**	**37 (43.5)**	

Data are presented as median [interquartile range], mean (standard deviation) or n (%).

^a^ Atheromatous vascular disease

^b^ Angiotensin converting enzyme inhibitors

^c^ Angiotensin II receptor blockers

^d^ Erythropoiesis stimulating agents

^e^ Calcium

^f^ Common carotid artery intima media thickness

Multivariate analysis (Table 4) showed that baseline presence of at least one carotid plaque, older age, and higher uric acid and cholesterol levels associated with an increased risk of plaque progression during follow-up. We detected a significant interaction between baseline presence of plaques and age, indicating that age predicted this outcome only in patients without plaques at baseline. Remarkably, the modality of dialysis did not predict progression of carotid plaques during follow-up.

**Table 4:** Multivariate logistic model for progression in plaques.

	OR	95%CI	p
Baseline presence of plaque	4.15	1.89–9.41	0.001
Age (year)	1.07	1.03–1.12	0.003
Baseline plaque/age interaction			0.019
Serum uric acid > 362.9μmol/L	2.56	1.22–5.66	0.016
Total cholesterol [3.91,4.81]mmol/L	0.63	0.25–1.58	0.47
Total cholesterol [4.81,10.7 mmol/L	0.32	0.12–0.84	0.023
**Dialysis modality (PD vs HD)**	**1.78**	**0.80–4.06**	**0.161**

### Analysis of survival to cardiovascular events

A total of 51 patients had a fatal or non fatal CV event (18 acute coronary syndromes, 10 ischaemic strokes, 8 limb ischaemia events, 6 sudden deaths, 4 hemorrhagic strokes, 2 mesenteric ischaemia events, 1 aortic aneurism rupture, 1 heart failure and 1 major arrhythmia) during a median follow-up of 21.36 months (range 0.16–53.91). Univariate analysis showed that factors predicting CV events were age (HR 1.02; p = 0.037); diabetic nephropathy as etiology of renal disease (HR 4.8; p<0.001) longer dialysis vintage (HR 0.98;p = 0.011), higher serum glucose levels (HR 1.01; p<0.001), treatment with antiplatelet drugs (HR 2.15; p = 0.005), baseline presence of carotid plaques (HR 5 p<0.001), baseline number of territories with plaque (HR 1.39; p<0.001), thicker cIMT (HR3.87; p = 0.007) and ischaemic ABI (HR 2.08; p = 0.024)

Multivariate analysis of survival to the first CV event (Table 5) identified smoking status, baseline presence of at least one carotid plaque, diabetic and vascular nephropathy and serum phosphate levels as independent predictors of this outcome. On the contrary, the modality of dialysis did not perform as a significant predictor of the risk of CV events.

**Table 5:** Multiple Cox regression analysis for incident CV events.

	HR	95%CI	p
Smokers (former and current)	2.07	1.10–3.89	0.023
Baseline presence of plaque	4.13	1.72–9.91	0.001
Etiology of renal disease:			
Diabetic nephropathy	4.33	2.20–8.50	<0.001
Vascular disease	1.50	0.66–3.37	0.327
Phosphate	1.29	1.04–1.59	0.019
**Dialysis modality (PD vs HD)**	**1.51**	**0.85–2.66**	**0.159**

## Discussion

The results of the present study showed that the modality of dialysis did not bear a differential impact either on the progression of atheromatous carotid or CV outcomes, when two relatively large samples of PD and HD patients, matched by age, gender, diabetes and dialysis vintage were compared, over a follow-up period of two years.

There is general agreement that ESRD patients suffer from accelerated atherosclerosis, as a consequence of the interplay of many traditional, uremia-related and novel risk factors [21]. The modality of dialysis can potentially influence the effects of some of these factors. For instance, both recurrent peritoneal loading with glucose-based dialysis solutions and a continuous peritoneal leak of proteins may result in a more atherogenic profile in PD patients, when compared with their counterparts on HD. The expected consequences include more severe degrees of dyslipidemia and insulin resistance in the former group. On the contrary, PD associates a better preservation of RKF, which may contribute to improve inflammation, endothelial dysfunction and vascular calcification, in these patients. However, the latter effects tend to fade with time on dialysis, as RKF declines. This can help to explain why in our study, which was restricted to prevalent patients, PD patients were more dyslipidemic, and presented worse blood pressure and mineral disease profiles than HD patients (Tables 1 and 2). Remarkably, these compared profiles did not match with the findings of baseline vascular US, which disclosed thinner cIMT and a lower proportion of patients with pathologic ABI values, in the PD group. The explanation for this apparent discrepancy is not clear, and could be a consequence of selection biases, but also of some protective effect of PD during the earlier phases of renal replacement therapy. In any case, atheromatous carotid disease progressed to a similar extent in both populations, and the incidence of CV events was not different. These findings support the current idea that PD and HD are medically equivalent, and that the selection of the modality of dialysis should be made according to the preference of the patient, after structured information and education processes [22].

The compared effect of the modality of dialysis on the progression of atherosclerotic disease has been insufficiently studied. A majority of the previous studies followed a cross-sectional design, and included relatively small samples of patients [11–13]. As a consequence the results were largely inconclusive. On the contrary, our study had a longitudinal design, and the study groups were matched for essential variables related to the preexistent atherosclerotic burden of the patients. This type of strategy brings the design of the study close to a randomized clinical trial [23], which is not feasible in practical terms, given the difficulties to randomize patients to the modality of dialysis [24]. This approach should be considered superior to multivariate Cox regression, at the time of correcting for confounding factors, in observational studies [23].

In agreement with the general results of the NEFRONA study [25], the baseline presence of any carotid plaque was a consistent predictor of progression of AD and CV events. Similar observations have been reported by some studies in HD patients [26–28]. In our study, AVD progression, including appearance of new plaques and CV events, was the rule in patients with carotid plaques at baseline, and the exception in those without. These findings support the utility of vascular US to detect CV risk subsets among ESRD patients with asymptomatic AD. This notwithstanding, the identification of phenotypic features could help to refine the identification of these subgroups.

Our results confirm the well-known association of some classic risk factors, including age and serum cholesterol, with AVD progression. Interestingly, we observed a statistical interaction between age and the baseline presence of carotid plaques, at the time of predicting the study outcomes. Older age predicted progression only in patients without plaques at baseline. This is in apparent contradiction with the notion that age is one of the most powerful correlates of AVD, and indicates that the presence of plaques in baseline US is more determinant, to predict outcomes. On the other hand, another apparently paradoxical finding was the inverse association between serum cholesterol levels and the progression of AVD. However, serum cholesterol has been claimed to present a U-shaped relationship to survival [29–31] and both, low and high levels, seem to have a negative impact. Serum uric acid levels presented a direct correlation with the risk of progression of AVD. Besides the general association with the genetic background, dietary intake and comorbid conditions, serum uric acid levels in ESRD patients are also dependent on increased degradation pathways, RKF, dialysis removal and drug therapies, among other factors. The association between uric acid and progression of AVD in patients with CKD has not been reported previously, other than by other analyses of the NEFRONA project [25]. However, some previous studies have suggested an association between hyperuricemia and the presence of carotid plaques in the general population [32] as well as in diabetic individuals [33] and in patients with an established diagnosis of CV disease [34]. Moreover, some ongoing studies are exploring the role of antihyperuricemic drugs on the progression of carotid atherosclerosis, as evaluated by US [35].

Smoking, diabetic nephropathy and serum phosphate levels showed an independent effect in the prediction of CV events. These findings are essentially confirmatory of current knowledge. For instance, there is evidence that smoking increases the CV risk of patients on dialysis [36,37]. The increased CV risk profile of diabetics all along the spectrum of CKD is also well-known, and was clearly detected in the baseline analysis of the NEFRONA study [38]. Finally, hyperphosphatemia has been clearly linked to the CV outcome of patients on dialysis [39,40].

This study has significant limitations. The conclusions cannot be applied to the overall population of patients on dialysis, because only individuals without known preexistent CV disease were subject of analysis. This potential selection bias is more likely for hemodialysis patients, because group matching restricted inclusion to those with similar characteristics to PD patients. The size of the sample was relatively large, but may still be considered insufficient, given the large amont of covariables as well as the high rate of drop-outs. The period of monitorization could also be considered too short, but the risk of bias linked to the high proportion of study drop-outs, particularly in the PD group, argued against a longer follow-up. Among the strengths of the study, we should mention the multicenter, prospective approach, the matched group design, the quality of the screening tools (including vascular US), and the presentation of clear conclusions, well supported by the results of the study.

In summary, atherosclerotic arterial disease is very prevalent among patients on dialysis, and progresses over time in a significant proportion of cases. The presence of vascular disease at baseline is the best individual predictor of progression. Our study was able to identify some demographic and clinical correlates of progression, including older age, smoking, diabetic nephropathy, and serum levels of cholesterol, uric acid and serum phosphate. Most importantly, the modality of dialysis did not appear to influence the progression of atherosclerotic disease. These results agree to the notion of an essential clinical equivalence of HD and PD for the management of ESRD, and support selection of the modality of dialysis based on informed decision by the patients.
