# Correction: The modality of dialysis does not influence atheromatous vascular disease progression or cardiovascular outcomes in dialysis patients without previous cardiovascular disease

**DOI:** 10.1371/journal.pone.0200226

**Published:** 2018-06-28

**Authors:** Mercè Borràs Sans, Miguel Pérez-Fontán, Montserrat Martinez-Alonso, Auxiliadora Bajo, Àngels Betriu, José M. Valdivielso, Elvira Fernández, on behalf of NEFRONA INVESTIGATORS

There are errors in the “Hemoglobin (mmol/L)” values in Table 1, Table 2 and Table 3. Please see the corrected tables here.

**Table 1:** Main Baseline characteristics according to dialysis modality.

	PD^a^ patients	HD^b^ patients	p
	(n = 237)	(n = 237)	
Males (%)	137 (58)	146 (61.8)	0.45
Age (years)	52 [42;63]	55 [43;63]	0.11
Smoker (former/current) (%)	135 (57)	137 (58)	0.92
Diabetes (%)	43 (18.1)	43 (18.1)	1
Hypertension (%)	219 (92.4)	199 (84)	0.007
Dyslipidemia (%)	153 (64.6)	123 (47.7)	<0.001
Etiology of renal disease (%):			0.670
Diabetic nephropathy	25 (10.5)	27 (11.4)	
Vascular disease	23 (9.7)	29 (12.2)	
Others	189 (79.7)	181 (76.4)	
Dialysis time (months)	13.7 [6.2;28.9]	14.3 [6.41;28.6]	0.91
Body mass index (kg/m^2^)	26.4 [23.3;29.2]	25.3 [23;28.6]	0.23
Systolic Blood Pressure (mmHg)	140 [129;159]	135 [120;151]	0.001
Diastolic Blood Pressure (mm Hg)	83 [77;94]	80 [70;88]	<0.001
Pulse Pressure (mmHg)	56 [45;69]	55 [46;63]	0.53
Serum glucose (mmol/L)	5.05 [2.96;6.10]	5.05 [4.56;6.10]	0.838
Urea (mmol/L)	21.5 [17.6;25.8]	20.8 [15.8;25]	0.07
Creatinine (μmol/L)	682.5 [519.8;884]	727.5 [574.6;884]	0.1
Total colesterol (mmol/L)	4.58 [3.99;5.20]	3.91 [3.37;4.53]	<0.001
HDL-cholesterol (mmol/L)	1.22 [1.01;1.48]	1.09 [0.90;1.29]	<0.001
LDL-cholesterol (mmol/L)	2.66 [2.07;5.15]	2.07 [1.63;2.64]	<0.001
Triglycerides (mmol/L)	1.34 [1.07;1.87]	1.41 [1.03;1.92]	0.75
Serum uric acid (μmol/L)	345.1 [303.4;401.6]	362.9 [321.3;425.4]	0.006
hs C-Reactive Protein (nmol/L)	19 [8.95;49.9]	22.47 [10.09;58.66]	0.23
Albumin (mol/L)	0.59 [0.54;0.62]	0.59 [0.54;0.64]	0.025
Hemoglobin (mmol/L)	7.2 [7.01;8.01]	7.26 [6.70;7.76]	<0.001
Corrected calcium (mmol/L)	2.3 [2.20;2.42]	2.26 [2.16;2.36]	<0.001
Phosphate (mmol/L)	1.6 [1.35;1.84]	1.54 [1.25;1.79]	0.035
iPTH (pmolL)	22.7 [14.9;35.4]	25.7 [14.3;36.4]	0.23
25-hydroxy-vitamin D (nmol/L)	28.9 [20.9;41.2]	37.4 [26.4;48.7]	<0.001
1-25-hydroxy-vitamin D (pmol/L)	13.6 [9.72;19.9]	13.8 [9.31;22.3]	0.54
Treatments			
Antihypertensive (%):	214 (90.3)	159 (67.1)	<0.001
ACEI^c^ (%)	66 (27.8)	41 (17.3)	0.008
ARBs^d^ (%)	111 (46.8)	54 (22.8)	<0.001
Diuretics (%)	128 (54)	45 (19)	<0.001
Statins (%)	137 (57.8)	110 (46.4)	0.017
Phosphate binders (%):	198 (83.5)	189 (79.7)	0.343
Binders without Ca^e^ (%)	126 (53.2)	138(58.2)	0.309
Binders with Ca^e^ (%)	42 (47.2)	40 (47.1)	1
Ca^e^ intake (binders) (gr/day)	1.5 [1;2]	1.65 [1;3]	0.027
Calcitriol/Paricalcitol (%)	97 (40.9)	122 (51.5)	0.027
Calcifedol /%)			
Cholecalciferol (%)	27 (11.4)	8 (3.38)	0.002
Cinacalcet (%)	11(4.64)	8 (3.38)	0.640
Antiplatelet drugs (%):	27 (24.1)	68 (28.7)	0.297
ESA^f^(%)	181(76.4)	198 (83.5)	0.066
Renal transplantation centre (%)	88 (37.1)	62 (26.2)	0.014
Plaque presence (%)	123 (51.9)	143 (60.3)	0.08
Number of territories with plaque	2 [1.0;3.0]	2 [1.0;3.0]	0.285
cIMT^g^ (mm)	0.65 [0.56;0.79]	0.70 [0.6;0.84]	0.009
Ankle-Brachial index (%)			
ABI < 0.9	37 (15.7)	23 (9.91)	0.08
ABI >0.9-<1.4	175 (74.5)	144 (62.1)	0.005
ABI > 1.4	23 (9.79)	65 (28)	<0.001

Data are presented as median [interquartile range], mean (standard deviation) or n(%).

^a^ Peritoneal dialysis

^b^ Hemodialysis

^c^ Angiotensin converting enzyme inhibitors

^d^ Angiotensin II receptor blockers

^e^ Calcium

^f^ Erythropoiesis stmulating agents

^g^ Common carotid artery intima media thickness

**Table 2:** Main Baseline characteristics of patients with 2-years follow-up according to dialysis modality.

	PD^a^ patients	HD^b^ patients	p
	(n = 82)	(n = 85)	
Males (%)	47 (57.3)	48 (52.2)	0.6
Age (years)	53 [42.5;63]	55 [44.8;64]	0.39
Smoker (former/current) (%)	49 (59.8)	48 (52.2)	0.9
Diabetes (%)	14 (17.1)	14 (15.2)	0.9
**Hypertension (%)**	75 (91.5)	79 (86)	0.359
**Dyslipidemia (%)**	58 (70.7)	55 (59.8)	0.176
Etiology of renal disease (%):			0.670
Diabetic nephropathy	7 (8.54)	8 (8.7)	
Vascular disease	8 (9.76)	13 (14.1)	
Others	67 (81.7)	71 (77.2)	
Dialysis time (months)	10.5 [4.46;19.2]	11.5 [5.82;25.8]	0.131
Body mass index (kg/m^2^)	26.4 [23.3;28.4]	26.1 [23;4.32.2]	0.446
Systolic Blood Pressure (mmHg)	145 (24.4)	138 (21.8)	0.03
Diastolic Blood Pressure (mm Hg)	87.5 (12.2)	78.8 (13.3)	<0.001
Pulse Pressure (mmHg)	56 [44;69]	56.5 [47;70.2]	0.579
Serum glucose (mmol/L)	5.16 [4.72;5.61]	5.23 [4.55;6.05]	0.931
Urea (mmol/L)	22.11 (6.76)	20.31 (6.13)	0.053
Creatinine (μmol/L)	596.7 [468.5;792.1]	676.3 [583.4;837.2]	0.815
Total colesterol (mmol/L)	4.75 [4.2;5.43]	3.9 [3.2;4.45]	0.258
HDL-cholesterol (mmol/L)	1.21 [1;1.52]	1.1 [0.88;1.21]	0.005
LDL-cholesterol (mmol/L)	3.46 [2.28;3.05]	3.36 [2.56;4.4]	<0.001
Triglycerides (mmol/L)	1.53 [1.09;2.02]	1.48 [1.13;1.94]	0.940
Serum uric acid (μmol/L)	350.4 (72.6)	369.4 (70.78)	0.093
hs C-Reactive Protein (nmol/L)	26.28 [10;61.24]	24 [10.47;62.48]	0.815
Albumin (mol/L)	0.59 [0.53;0.62]	0.59 [0.54;0.64]	0.471
Hemoglobin (mmol/L)	7.24 (0.80)	6.82 (0.84)	0.002
Corrected calcium (mmol/L)	2.32 [2.22;2.4]	2.25 [2.16;2.32]	0.031
Phosphate (mmol/L)	1.52[1.32;1.81]	1.55 [1.29;1.81]	0.965
iPTH (pmolL)	21.1 [15.48;34.25]	26.51 [16.2;41]	0.182
25-hydroxy-vitamin D (nmol/L)	30.5 [21.75;43.75]	35.5 [27;48]	0.014
1-25-hydroxy-vitamin D (pmol/L)	15.45 [10.3;22.7]	14.4 [10.15;25.75]	0.862
Treatments			
Antihypertensive (%):	72 (87.8)	60 (65.2)	0.001
ACEI^c^ (%)	20 (24.4)	15 (16.3)	0.255
ARBs^d^ (%)	37 (45.1)	25 (27.2)	0.021
Diuretics (%)	27 (32.6)	28 (32.9)	0.837
Statins (%)	48 (58.3)	50 (54.3)	0.687
Phosphate binders (%):	66 (80.5)	68 (73.9)	0.396
Binders without Ca^e^ (%)	38 (46.3)	48(52.2)	0.538
Binders with Ca^e^ (%)	42 (47.2)	40 (47.1)	1
Ca^e^ intake (binders) (gr/day)	1 [1;1.5]	1.5 [1;2.5]	0.03
Calcitriol/Paricalcitol (%)	34 (41.5)	44 (47.8)	0.490
Calcifedol /%)	8 (9.76)	5 (5.43)	0.428
Cholecalciferol (%)	2 (2.44)	4 (4.35)	0.685
Cinacalcet (%)	18 (22)	24 (26.1)	0.646
Antiplatelet drugs (%):	22 (26.8)	27 (29.3)	0.842
ESA^f^ (%)	62 (75.6)	77 (83.7)	0.255
Renal transplantation centre (%)	27 (32.9)	20 (21.7)	0.137
Plaque presence (%)	44 (53.7)	53 (57.6)	0.711
Number of territories with plaque	2 [1.0;2.0]	2 [1.0;3.0]	0.149
cIMT^g^ (mm)	0.65 [0.57;0.75]	0.72 [0.62;0.86]	0.005
Ankle-Brachial index (%)			<0.001
ABI <0.9	10 (12.2)	7 (7.78)	
ABI >0.9-<1.4	66 (80.5)	55 (61.1)	
ABI > 1.4	6 (7.32)	21 (31.1)	

Data are presented as median [interquartile range], mean (standard deviation) or n (%).

^a^ Peritoneal dialysis

^b^ Hemodialysis

^c^ Angiotensin converting enzyme inhibitors

^d^ Angiotensin II receptor blockers

^e^ Calcium

^f^ Erythropoiesis stimulating agents

^g^ Common carotid artery intima media thickness

**Table 3:** Main Baseline characteristics of patients with 2-years follow-up according to dialysis modality.

	AVD^a^ progression	Non AVD progression	p
	(n = 89)	(n = 85)	
Males (%)	50 (56.2)	45 (52.9)	0.78
Age (years)	57 [48;65]	50 [39;62]	0.004
Smoker (former/current) (%)	53 (59.6)	44 (51.8)	0.38
Diabetes (%)	20 (22.5)	8 (9.41)	0.033
Hypertension (%)	79 (88.8)	75 (88.2)	1
Dyslipidemia (%)	59 (66.3)	54 (63.5)	0.824
Etiology of renal disease (%):			0.165
Diabetic nephropathy	25 (10.5)	27 (11.4)	
Vascular disease	23 (9.7)	29 (12.2)	
Others	69 (77.5)	69 (81.2)	
Dialysis time (months)	10.2 [4.4;23.2]	11.8 [6.47;22.5]	0.27
Body mass index (kg/m^2^)	27 [23.6;30.7]	26.1 [23.1;28.8]	0.156
Systolic Blood Pressure (mmHg)	142 (24.3)	141 (24.3)	0.735
Diastolic Blood Pressure (mm Hg)	82 (13.5)	83.9 (13.5)	0.343
Pulse Pressure (mmHg)	60 [44;74]	56 [45;66]	0.326
Serum glucose (mmol/L)	5.18 [4.54;6.10]	5.05 [4.49;5.66]	0.157
Urea (mmol/L)	18.3 (5.25)	18.3 (5.21)	0.984
Creatinine (μmol/L)	610 [486.2;797.4]	662.1 [557.8;839.8]	0.089
Total colesterol (mmol/L)	4.14 [3.63;4.89]	4.45 [3.57;5.33]	0.258
HDL-cholesterol (mmol/L)	1.10 [0.93;1.39]	1.16 [0.96;1.37]	0.561
LDL-cholesterol (mmol/L)	2.30 [1.85;2.82]	2.61 [1.81;2.98]	0.264
Triglycerides (mmol/L)	1.51 [1.11;2.10]	1.38 [1.07;1.92]	0.528
Serum uric acid (μmol/L)	370.5 (72.6)	350.4 (70.8)	0.084
hs C-Reactive Protein (nmol/L)	37.7 [13.8;65.9]	18.5 [8.4;54.2]	0.04
Albumin (mol/L)	0.59 [0.0.53;0.63]	0.59 [0.54;0.62]	0.613
Hemoglobin (mmol/L)	7 (0.95)	7 (0.80)	0.962
Corrected calcium (mmol/L)	2.31 [2.19;2.4]	2.26 [2.12;2.37]	0.419
Phosphate (mmol/L)	1.52 [1.26;1.81]	1.55 [1.36;1.84]	0.292
iPTH (pmolL)	24.7 [15.9;38.1]	23.3 [15.27;36.7]	0.586
25-hydroxy-vitamin D (nmol/L)	30.9 [22.8;42.9]	36.4 [26.7;48.7]	0.043
1-25-hydroxy-vitamin D (pmol/L)	14.9 [10.0;24.5]	13.6 [9.7;22.3]	0.602
Treatments			
Antihypertensive (%):	68 (76.4)	64 (75.3)	1
ACEI^b^ (%)	18 (20.2)	17 (20)	1
ARBs^c^ (%)	29 (32.6)	33 (38.8)	0.483
Diuretics (%)	27 (32.6)	28 (32.9)	0.837
Statins (%)	51 (57.3)	47 (55.3)	0.9
Phosphate binders (%):	68 (76.4)	66 (77.6)	0.98
Binders without Ca^d^ (%)	39 (43.8)	47 (55.3)	0.173
Binders with Ca^d^ (%)	42 (47.2)	40 (47.1)	1
Ca^d^ intake (binders) (gr/day)	1 [1;1.5]	1.5 [1;2.5]	
Calcitriol/Paricalcitol (%)	45 (50.6)	33 (38.8)	0.16
Calcifedol /%)	9 (10.1)	4 (4.71)	0.286
Cholecalciferol (%)	3 (3.37)	3 (3.53)	1
Cinacalcet (%)	19 (21.3)	23 (27.1)	0.482
Antiplatelet drugs (%):	27 (30.3)	22 (25.9)	0.628
ESA^e^ (%)	66 (74.2)	73 (85.9)	0.082
Renal transplantation centre (%)	22 (24.7)	25 (29.4)	0.599
Plaque at baseline (%)	64 (71.9)	33 (33.8)	<0.001
Number of territories with plaque	2 [1.0;3.0]	2 [1.0;3.0]	0.398
cIMT^f^ (mm)	0.73 [0.65;0.89]	0.64 [0.56;0.74]	<0.001
Ankle-Brachial index (%)			
ABI <0.9	11 (12.5)	6 (7.14)	0.357
ABI >0.9-<1.4	57 (64.8)	64 (76.2)	0.141
ABI > 1.4	20 (22.7)	14 (16.7)	0.420
**Dialysis modality:**			**0.437**
**Hemodialysis**	**44 (49.4)**	**48 (56.5)**	
**Peritoneal dialysis**	**45 (50.6)**	**37 (43.5)**	

Data are presented as median [interquartile range], mean (standard deviation) or n (%).

^a^ Atheromatous vascular disease

^b^ Angiotensin converting enzyme inhibitors

^c^ Angiotensin II receptor blockers

^d^ Calcium

^e^ Erythropoiesis stimulating agents

^f^ Common carotid artery intima media thickness
